# Apigetrin ameliorates doxorubicin prompted testicular damage: biochemical, spermatological and histological based study

**DOI:** 10.1038/s41598-024-59392-x

**Published:** 2024-04-20

**Authors:** Muhammad Umar Ijaz, Saba Yaqoob, Ali Hamza, Mehwish David, Tayyaba Afsar, Fohad Mabood Husain, Houda Amor, Suhail Razak

**Affiliations:** 1 Department of Zoology, Wildlife and Fisheries, University of Agriculture, Faisalabad, 38040 Pakistan; 2 Department of Animal Sciences, Quaid-I-Azam University, Islamabad, 45320 Pakistan; 3 Department of Community Health Sciences, College of Applied Medical Sciences, King Saud University, P.O. Box 2455, 11451 Riyadh, Saudi Arabia; 4 Department of Food Science and Nutrition, College of Food and Agriculture Sciences, King Saud University, Riyadh, Saudi Arabia; 5 Department of Obstetrics, Gynecology and Reproductive Medicine, Saarland University Clinic, Homburg, Germany

**Keywords:** Doxorubicin, Apigetrin, Oxidative stress, Testicular damage, Apoptosis, Biochemistry, Zoology

## Abstract

Doxorubicin (DOX) is a highly effective, commonly prescribed, potent anti-neoplastic drug that damages the testicular tissues and leads to infertility. Apigetrin (APG) is an important flavonoid that shows diverse biological activities. The present research was designed to evaluate the alleviative role of APG against DOX-induced testicular damages in rats. Forty-eight adult male albino rats were randomly distributed into 4 groups, control, DOX administered (3 mgkg^−1^), DOX + APG co-administered (3 mgkg^−1^ of DOX; 15 mgkg^−1^ of APG), and APG administered group (15 mgkg^−1^). Results of the current study indicated that DOX treatment significantly reduced the activities of superoxide dismutase (SOD), glutathione reductase (GSR), catalase (CAT) and glutathione peroxidase (GPx), while increasing the levels of malondialdehyde (MDA) and reactive oxygen species (ROS). DOX treatment also reduced the sperm count, viability, and motility. Moreover, DOX significantly increased the sperm morphological anomalies and reduced the levels of plasma testosterone, luteinizing hormone (LH) and follicle-stimulating hormone (FSH). The administration of DOX significantly increased the expressions of Bax and Caspase-3, as well as the levels of inflammatory markers. Additionally, DOX treatment significantly downregulated the expressions of steroidogenic enzymes (StAR, 3β-HSD and 17β-HSD) and Bcl-2. Furthermore, DOX administration provoked significant histopathological abnormalities in the testicular tissues. However, APG supplementation significantly reversed all the testicular damages due to its androgenic, anti-apoptotic, anti-oxidant and anti-inflammatory nature. Therefore, it is concluded that APG may prove a promising therapeutic agent to treat DOX-induced testicular damages.

## Introduction

Chemotherapy is a commonly prescribed cancer treatment that refers to the use of chemicals agents to destroy cancerous cells. It is considered to be highly effective in combating cancer^1^_._ The long-term use of anti-tumor drugs can cause serious toxic effects that may damage the body, which ultimately restricts their clinical application^2^. Unfortunately, the incidence of cancer has been reached to the point where it is considered to be the second leading cause of death worldwide, followed by heart disease^3^. Chemotherapeutic advancements have increased the life expectancy of cancer patients, 82% of cancer patients are currently enjoying the expectancy of life up to 5 years due to progressive advancements in chemotherapy^4^. Cancer is a serious public health challenge that causes profound morbidity and mortality globally^5^_._ DOX can induce hepatotoxicity, nephrotoxicity^6,7^ as well as testicular toxicity^8^.

According to the previous literature, DOX has the potential to induce oxidative stress (OS), lipid peroxidation (LP) and testicular damage^9^. The oxidative damage induced by DOX treatment is due to a combination of free radicals that contain superoxide, hydroxyl radicals and iron. These free radicals disrupt the mitochondrial membrane present in the sperm mid-piece and result in reduced ATP production^10^. DOX has the potential to reduce sperm quantity, motility and increase sperm abnormalities in rats^11^.

Natural products are the primary source of the active ingredients of medicines. More than 80% medicines are derived from natural sources^12^. Plant-based bioactive substances, such as flavonoids have received a lot of attention during the last few years due to their broad range of pharmacological effects, such as antioxidant, anti-apoptotic, and anti-inflammatory. Plants use flavonoids to protect themselves from biotic and abiotic stress and to defend against plaques^13^. They are considered as risk-free and show little adverse effects^14,15^. Apigetrin (APG) is an important naturally occurring flavonoid^16^ that is extracted from plant leaves, seeds, vegetables, fruits^17^ and several herbal medicines including *Teucrium gnaphalodes, Stachys tibetica Vatke,* and *Scutellaria baicalensis Georgi*^18^. APG shows anti-fungal, anti-oxidant, anti-mutagenic, anti-inflammatory^19^ and anti-cancer properties^20^. To overcome DOX induced testicular toxicity, a detailed investigation on plant-based remedies is required. Therefore, the present study was designed to evaluate the ameliorative role of APG against DOX-induced testicular damage in rats.

## Materials and methods

### Chemicals

Doxorubicin (DOX) (Physical form: Liquid; CAS number: 25316–40-9; Molecular weight: 579.98) and apigetrin (APG) (Physical form: Solid; CAS number: 578–74-5; Molecular weight: 432.38) were obtained from *Sigma Aldrich* (Germany).

### Animals

In the current experiment, adult male albino rats (250 ± 20 g) were used. The rats were placed in steel cages maintained at standard temperature (22–25°C), 12-h dark/light cycle and humidity (45 ± 5%) in the animal house of the University of Agriculture, Faisalabad (UAF). The animals were provided free access to commercial food and water and the protocol of the study was approved by UAF ethical committee of Animal Protection and Handling. The study procedures were performed by following the ARRIVE guidelines^21^ and all the methods were performed in accordance with the relevant guidelines and regulations.

### Experimental protocol

Fourty eight rats were distributed into 4 groups, 12 rats per group. First group was designed as a control group. Second group was administered with DOX (3 mg/kg), Third group was co-administered with DOX (3 mg/kg) and APG (15 mg/kg) and the Forth group was administered with APG (15 mg/kg) only. The 3 doses of DOX were administered intraperitoneally after every 2 weeks, whereas the dose of APG was administered daily through oral gavage. The dose of DOX was selected according to the previous study^22^, while APG dose was selected in compliance with the study of Guo et al.^23^ After the completion of 56 days of treatment, the rats were anesthetized using combined intraperitoneal injection of ketamine (75 mg/kg) and xylazine (2.5 mg/kg)^24^. Anesthetized rats were secured in a supine position and a thoracotomy was performed to assure euthanasia. The euthanasia method was performed in accordance with AVMA guidelines^25^. The blood was collected in sterile containers and serum samples were obtained by centrifuging blood at 1000 g for 15 min and stored at − 20 $^\circ{\rm C}$ for biochemical assays. Testes were removed, right testis was stored in zipper bags for biochemical observation at − 80 °C, testicular tissues were homogenized in Na_3_PO_4_ buffer solution at 12,000 rpm for 14–15 min. Finally, various parameters were evaluated by using the supernatant. The right testis was fixed in formalin (10%) for histopathological analysis.

### Assessment of catalase (CAT)

The activity of CAT in was determined in accordance with the technique of Chance and Maehly^26^. 2.5 mL of 50 mM phosphate buffer (pH 5.0), 0.4 mL of 5.9 mM H_2_O_2_, and 0.1 mL enzyme extract were mixed to make reaction mixture. Absorbance changes in the mixture were observed at 240 nm. One unit of CAT activity was considered as an absorbance change of 0.01 as units/min.

### Assessment of superoxide dismutase (SOD)

The activity of SOD was assessed by using the method of Kakkar et al.^27^ Reaction mixture consisted of 1.2 mL of sodium pyrophosphate buffer (0.052 mM; pH 7.0) and 0.1 mL of phenazine methosulphate (186 mM). 0.3 mL of supernatant after centrifugation (1500 × *g* for 10 min followed by 10,000 × g for 15 min) of homogenate was added to the reaction solution. Then, 0.2 mL of NADH (780 mM) was added to initiate enzyme reaction, which was later stopped by adding 1 mL of glacial acetic acid. Finally, chromogen’s amount was assessed by noticing the change in color intensity (at 560 nm). The values of SOD activity were presented as unit/mg protein.

### Assessment of glutathione peroxidase (GPx)

The activity of GPx was evaluated according to the protocol of Rotruck et al.^28^ Reaction mixture of GPx activity consisted of 0.01 mL of 10 mM sodium azide, 2.0 mL of 0.4 M Tris–HCl buffer (pH 7.0), 0.5 mL of 0.2 mM. H_2_O_2_ and 0.2 mL of 10 mM glutathione. Incubation was performed at 37 °C for about 10 min. and then 0.4 mL 10% (*v*/v) TCA was added for termination of reaction. The mixture was centrifuged at 5000 rpm for about 5 min. and absorbance was noticed at 430 nm. Its final values were shown as unit/mg protein.

### Assessment of glutathione reductase (GSR)

The activity of GSR was estimated according to the procedure of Carlberg and Mannervik^29^. Reaction mixture consisted of 0.1 mL EDTA (0.5 mM), 0.05 mL oxidized glutathione (1 mM), 0.1 mL NADPH (0.1 mM), 1.65 mL phosphate buffer (0.1 M, pH 7.6) and 0.1 mL of 10% homogenate in a volume of 2 mL. Enzymatic activity was measured at 25 °C by noticing NADPH disappearance at about 340 nm. The values obtained were presented as nM NADPH oxidized/min/mg tissue.

### Assessment of MDA level

MDA level was measured by the method of Afsar et al.^30^ The quantification of thiobarbituric acid reactive substances was assessed by comparing the absorption to the standard curve of MDA equivalents generated by acid-catalyzed hydrolysis of 1, 1, 3, 3 tetramethoxy propane. The values of MDA were displayed as nmoL/mg protein.

### Assessment of ROS level

ROS level was determined by 2′, 7′ -dichlorofluorescein diacetate (DCFH-DA). In brief, 100 μL cell lysates or renal homogenate were reacted with 100 μL 2 mg/mL DCFH-DA. A fluorescence plate reader was employed to monitor the fluorescence change after 30 min incubation at 37 ◦C. The excitation and emission wavelengths were respectively set at 488 nm and 525 nm. Result was displayed as relative fluorescence unit (RFU) per mg protein.

### Semen analysis

The caudal portion of the epididymis was used to collect sperm samples. The caudal portion was minced and incubated in physiological saline for 30 min at 37 °C to release the spermatozoa. Then sperm motility was observed under a phase-contrast microscope (400X)^31^. Eosin or nigrosin staining and microscopic examination were used to evaluate the sperm viability. Moreover, epididymal sperm count was counted using a hemocytometer^32^. The sperm structural anomalies (head, tail and midpiece) were evaluated by using the Filler method^33^.

### Hypo-osmotic swelling test

HOS test was used to determine the sperm integrity by following the Correa and Zavos^34^ protocol. The current experiment was performed by mixing 180 μL of fructose solution with 20 μL of semen sample by keeping the 80 mOsm/L osmotic pressure for twenty minutes. Following incubation, sperms were stained using eosin or nigrosin. Moreover, 200 sperm of swollen and non-swollen tails were calculated at 400X.

### RNA extraction and real-time quantitative reverse transcription-polymerase chain reaction (qRT-PCR)

Steroidogenic enzymes and apoptotic markers expressions were evaluated by using RT-qPCR. The tissue samples were homogenized by employing a rotor stator homogenizer to isolate RNA. After centrifugation, the supernatant was collected and 70% ethanol was added. RNA spin columns were used for purification. DNase I treatment was accomplished, followed by multiple washes. Ethanol was removed and the isolated RNA was stored at –70 °C. RNA quality was determined with the help of NanoDrop Spectrophotometer, using RNA samples having A260/A280 ratio between 1.8 and 2.0. cDNA was synthesized from RNA samples using the Thermo Scientific Revert Aid RT Kit and reverse transcriptase enzyme. RT-qPCR was carried out in a LightCycler 480 instrument (Roche, Basel, Switzerland) using Syber green PCR master mix (TaKaRa, Biotech. Co., Ltd.). PCR amplification was started by denaturation for 3 min at 95 °C that was followed by 45 cycles of 95 °C for 10 s, 60 °C for 40 s, 72 °C for 32 s and a final incubation at 75 °C for 5 min. β-actin was considered as internal control and 2^−ΔΔCT^ was used to assess the variations in the expressions of these parameters. Table 1 displays the primer sequences of the target genes as reported previously^35^.

**Table 1:** Primers sequences for the real-time quantitative reverse transcription-polymerase chain reaction.

Gene	Primers 5’—3’	Accession number	Product size
3β-HSD	Forward: GCCACCCTTTAACTGCCACT	NM_0010079	119
	Reverse: CTGTGCTGCTCCACTAGTGT		
17β-HSD	Forward: TATCCAGGTGCTGACCCCTT	NM_054007	103
	Reverse: CAAGGCAGCCACAGGTTTCA		
StAR	Forward: AGCGTAGAGGTTCCACCTGT	NM_031558	123
	Reverse: ATACTGAGCAGCCACGTGAG		
Bax	Forward: GCACTAAAGTGCCCGAGCTG	NM_017059.2	148
	Reverse: CCAGATGGTGAGTGAGGCAG		
Bcl-2	Forward: ACTGAGTACCTGAACCGGCA	NM_016993.1	139
	Reverse: CCCAGGTATGCACCCAGAGT		
Caspase-3	Forward: GTACAGAGCTGGACTGCGGT	NM_012922.2	137
	Reverse: TCAGCATGGCGCAAAGTGAC		
β-actin	Forward: AGGAGATTACTGCCCTGGCT	NM_031144	138
	Reverse: CATTTGCGGTGCACGATGGA		

3β-hydroxysteroid dehydrogenase (3β-HSD); 17β-hydroxysteroid dehydrogenase (17β-HSD); Steroidogenic acute regulatory protein (StAR).

### Hormonal analysis

Plasma testosterone (serial number H090), LH (serial number H206) and FSH (serial number H101) levels were measured using (ELISA) kits by following the company’s directions (Los Angeles, CA, USA). The 96-well ELISA plate was filled with assay diluent (50 µL) as well as plasma (10 µL). It was then incubated at room temperature for two hours. Following a rinse with deionized water, the plates were incubated for a maximum of two hours before 100 µL of peroxidase-conjugated immunoglobulin G (IgG) anti-LH, anti-FSH or anti-testosterone solution was added to each well. Plates were once again washed with deionized water, then wells were filled with substrate solution and incubated for 25 min at room temperature. To halt the reaction, stop solution (50 µL) was added. Lastly, at 450 nm, the absorbance of plasma testosterone, LH, and FSH was measured. To prevent inter-assay variance, each sample was conducted in triplicate under the same circumstances at the same time^36^.

### Inflammatory indices

The levels of NF-κΒ (CSB-E13148r), TNF-α (CSB-E07379r), IL-1β (CSB-E08055r) and IL-6 (CSB-E04640r) and COX-2 (CSB-E13399r) activity were evaluated by ELISA kits (Cusabio Technology Llc, Houston, TX, USA) and the instructor’s guidelines were followed. Firstly, 50 µL of sample was dispensed to the microplate wells. After that, 50 µL of antibody cocktail was poured to the wells. Plates were incubated at room temperature for the duration of 1 h. After washing properly with the help of wash buffer, 100 µL of TMB substrates were dispensed to each well and incubated for about 10 min. After the addition of 100 µL of stop solution, the color was developed. The optical density was noted at 450 nm using Tecan Multimode Reader^36^.

### Histopathology

The tissues were kept in 10% formalin after cleaning with a normal saline solution. The tissues were gradually dehydrated by using the rising grades of alcohol 70%, 90% and 100% and then fixed in paraffin wax. 4–5-µm tissue slices were cut with the help of a microtome and stained with hematoxylin–eosin. Then, these slides were examined under a light microscope (400X) and microphotography was carried out using a Leica LB microscope connected to a camera. The photos were analyzed by using Image J2X software.

### Statistical analysis

Data were presented as mean ± SEM. One-way ANOVA followed by Tukey's test was performed by using Minitab software. The level of significance was set at *P* < 0.05.

### Ethical approval

The protocol of the study was approved by UAF ethical committee of Animal Protection and Handling. The study procedures were performed by following the ARRIVE guidelines and all the methods were performed in accordance with the relevant guidelines and regulations.

## Results

### Effect of DOX and APG on biochemical parameters

In the current study DOX administration induced a significant (*P* < 0.001) decrease in the activities of GPx, GSR, CAT and SOD. On the other hand, a significant (*P* < 0.001) increase in the levels of MDA and ROS was observed as compared to control group. However, the co-administration of APG + DOX significantly (*P* < 0.01) increased the activities of GPx, GSR, CAT, and SOD. Moreover, MDA and ROS levels were significantly (*P* < 0.01) reduced in co-administered group as compared to DOX-administered group. Moreover, in APG only administered group these parameters were comparable to the control group (Table 2).

**Table 2:** Effect of DOX and APG on biochemical parameters.

Parameters	Groups			
	Control	DOX	DOX + APG	APG
CAT (Umg^−1^ protein)	9.35 ± 0.29	4.94 ± 0.12^###^	7.53 ± 0.58**	9.38 ± 0.30***
SOD (Umg^−1^ protein)	7.02 ± 0.17	3.35 ± 0.14^###^	5.28 ± 0.18**	7.07 ± 0.12***
GPx (Umg^−1^ protein)	23.73 ± 0.99	7.19 ± 0.21^###^	18.42 ± 0.73**	23.79 ± 0.98***
GSR (nM NADPH oxidized/min/mg tissue)	6.31 ± 0.28	1.48 ± 0.14^###^	4.77 ± 0.10**	6.35 ± 0.29***
ROS (RFU/mg protein)	0.72 ± 0.08	8.12 ± 0.28^###^	2.54 ± 0.24**	0.68 ± 0.09***
MDA (nmol/mg protein)	0.69 ± 0.14	6.06 ± 0.17^###^	1.51 ± 0.13**	0.66 ± 0.07***

Values based on mean ± SEM values. Significant differences are displayed as ^###^*P* < 0.001 compared to control group; **P < 0.01 compared to DOX-administered group, ***P < 0.001 compared to control group.

### Effect of DOX and APG on spermatogenic parameters

DOX-intoxication prompted a significant (*P* < 0.01) reduction in the sperm viability, count and motility, whereas a significant (*P* < 0.01) increase in the dead sperm count and sperm structural anomalies (tail, head and mid-piece) were observed as compared to control. However, APG + DOX co-administration significantly (*P* < 0.01) reduced the sperm abnormalities induced by DOX and significantly (*P* < 0.01) increased the sperm motility, viability and sperm number in comparison to DOX administered group. Moreover, these sperm indices in APG alone administered group were close to the control group (Table 3).

**Table 3:** Effect of DOX and APG on sperm parameters.

Parameters	Groups			
	Control	DOX	DOX + APG	APG
Epididymal sperm count (million/mL)	25.68 ± 1.21	7.33 ± 0.53^##^	18.36 ± 0.77**	25.93 ± 1.33***
Motility (%)	81.97 ± 1.22	33.84 ± 0.93^##^	72.37 ± 1.01**	82.56 ± 1.66***
Dead sperms (%)	15.92 ± 0.93	75.78 ± 0.87^##^	34.24 ± 1.19**	14.89 ± 1.03***
Head abnormality (%)	2.74 ± 0.37	33.38 ± 1.20^##^	7.41 ± 0.73**	2.69 ± 0.38***
Mid sperm abnormality (%)	0.58 ± 0.08	13.37 ± 1.27^##^	2.63 ± 0.19**	0.55 ± 0.08***
Tail abnormality (%)	4.71 ± 0.53	23.12 ± 1.89^##^	9.88 ± 0.45**	4.69 ± 0.52***
Hypo- osmotic swelled sperm count (HOS) (%)	87.92 ± 2.28	25.35 ± 1.15^##^	71.63 ± 0.83**	89.08 ± 2.38***

Values based on mean ± SEM values. Significant differences are displayed as ^##^*P* < 0.01 compared to control group; **P < 0.01 compared to DOX-administered group, ***P < 0.001 compared to control group.

### Effect of DOX and APG on steroidogenic enzymes

**Figure 1:**
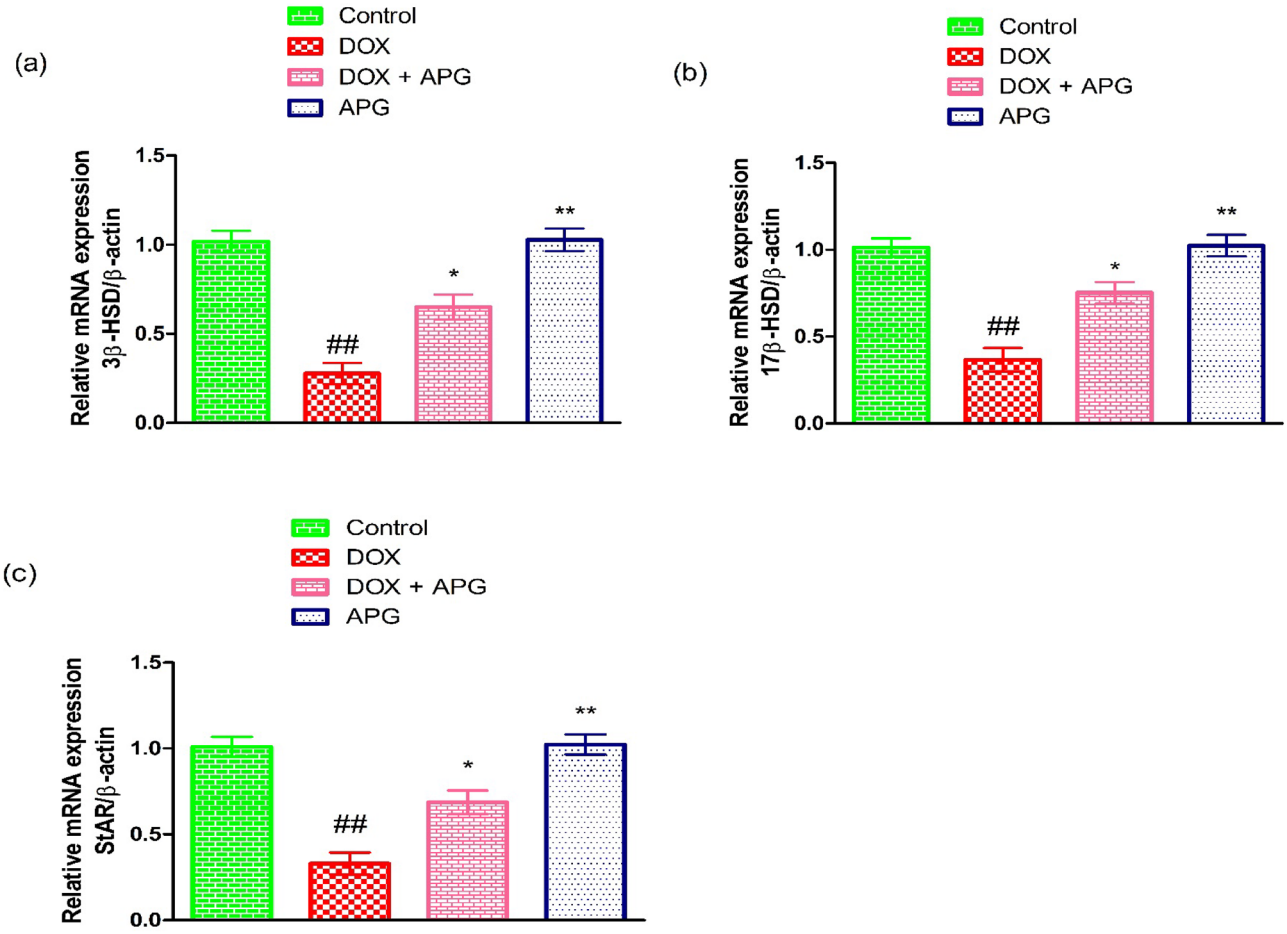
Effect of DOX and APG on the expression of (**a**) 3β-HSD, (**b**) 17β-HSD and (**c**) StAR. The bars are based on mean ± SEM values. Significant differences are displayed as ^##^*P* < 0.01 compared to control group; ^*^P < 0.05 compared to DOX-administered group, ^**^P < 0.01 compared to control group.

DOX-intoxication led to a significant (*P* < 0.01) decrease in the expressions of steroidogenic enzymes (17β-HSD, 3β-HSD and StAR) in contrast to the control group. Nonetheless, the co- administration of APG + DOX significantly (*P* < 0.05) increased these expressions in contrast to DOX-administered group. Moreover, the expressions of these steroidogenic enzymes in only APG administered group were close to the control group (Fig. 1).

### Effect of DOX and APG on hormones

**Figure 2:**
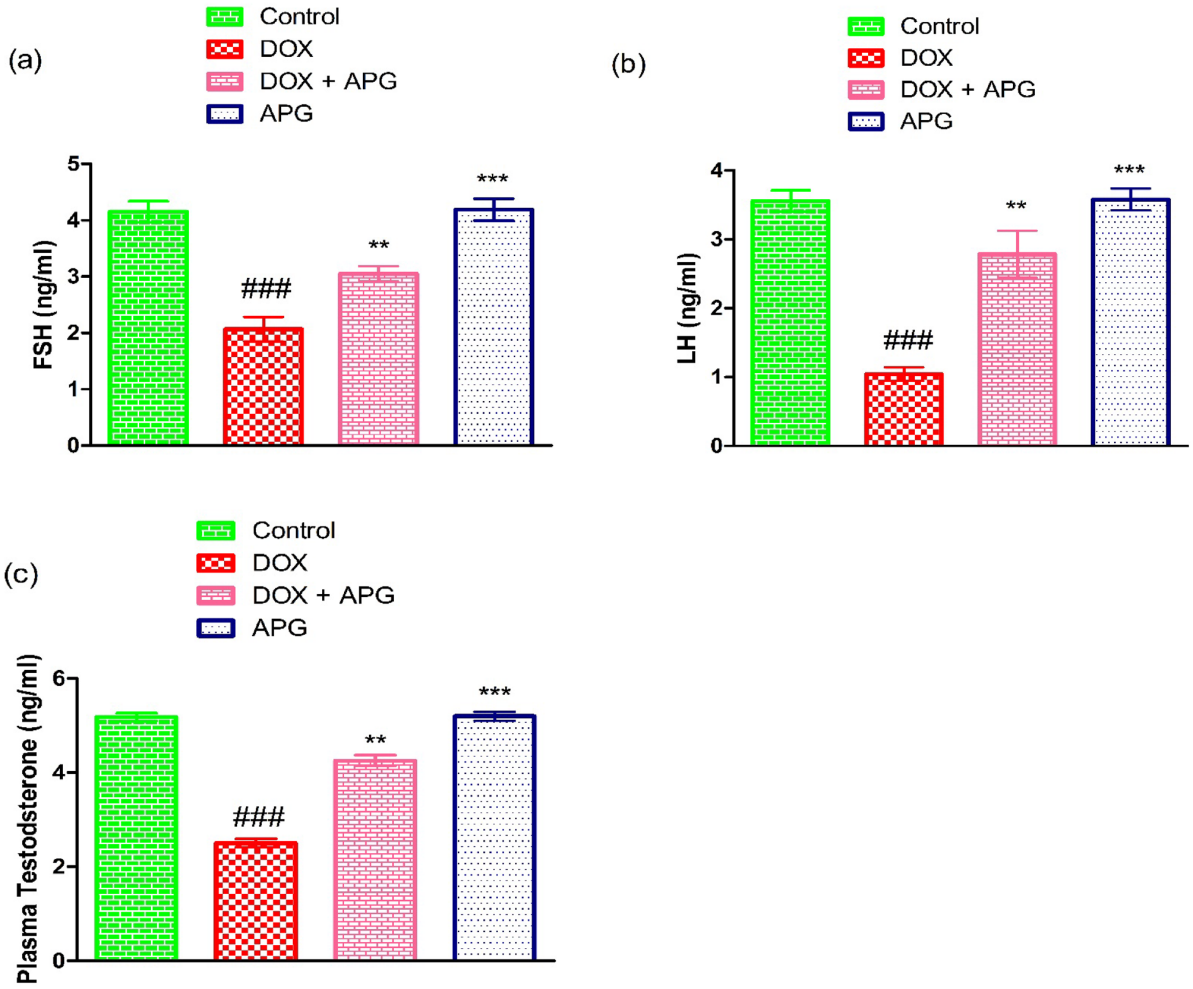
Effect of DOX and APG on the level of (**a**) FSH, (**b**) LH and (**c**) Plasma testosterone. The bars are based on mean ± SEM values. Significant differences are displayed as ^###^*P* < 0.001 compared to control group; ^**^P < 0.01 compared to DOX-administered group, ^***^P < 0.001 compared to control group.

DOX exposure significantly (*P* < 0.001) decreased the levels of LH, FSH and testosterone in contrast to the control group. However, co-administration of APG + DOX resulted in a considerable (*P* < 0.01) increase in the hormonal level in contrast to DOX-administered group. Moreover, in only APG administered group the hormonal level was close to the control group (Fig. 2).

### Effect of DOX and APG on apoptotic markers

**Figure 3:**
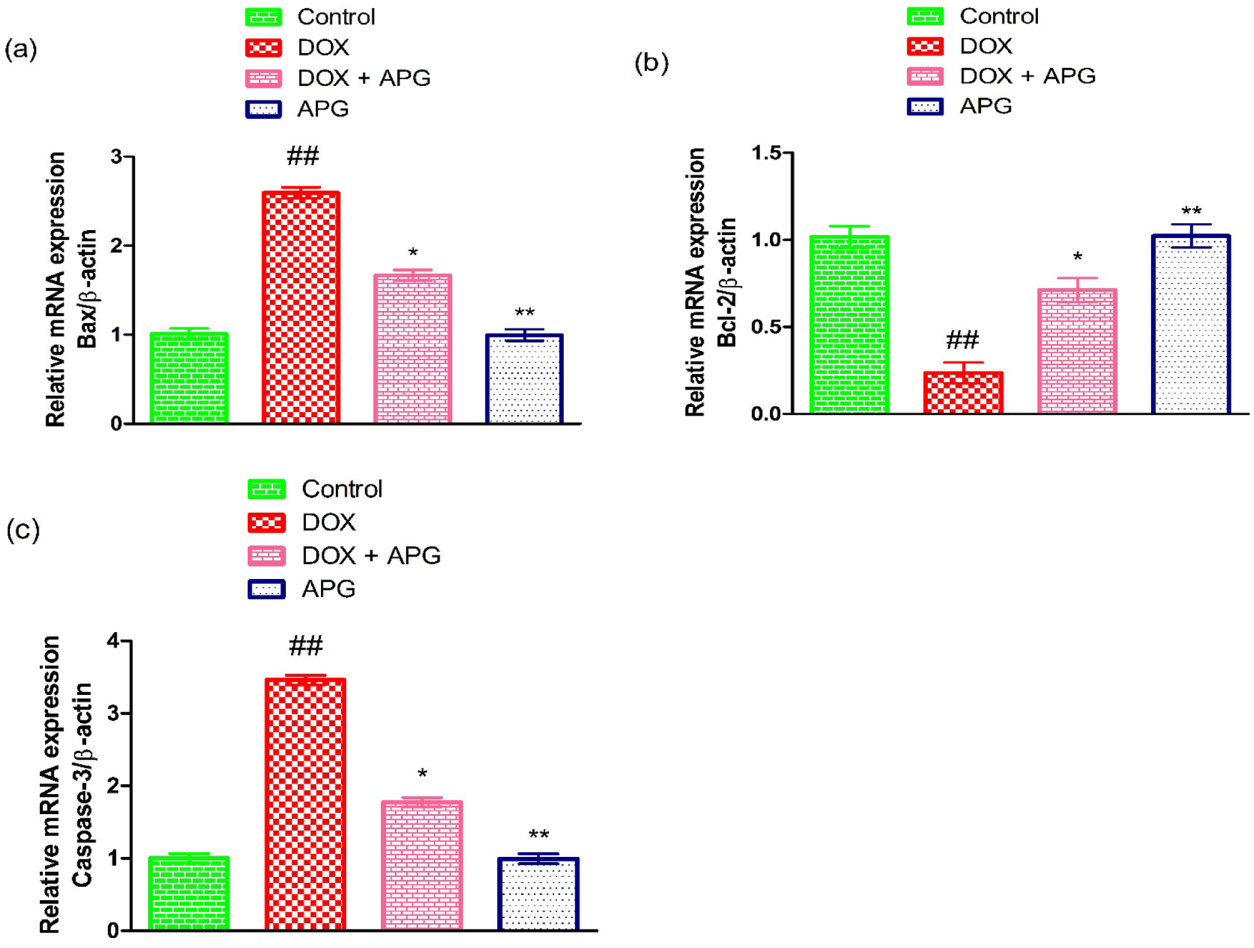
Effect of DOX and APG on the expression of (**a**) Bax, (**b**) Bcl-2 and (**c**) Caspase-3. The bars are based on mean ± SEM values. Significant differences are displayed as ^##^*P* < 0.01 compared to control group; ^*^P < 0.05 compared to DOX-administered group, ^**^P < 0.01 compared to control group.

DOX-intoxication resulted in a significant (*P* < 0.01) reduction in the expression of Bcl-2, while a significant (*P* < 0.01) upsurge in the expressions of Caspase-3 and Bax as compared to control group. However, the co-administration of APG + DOX considerably (*P* < 0.05) decreased Caspase-3 and Bax expressions, while significantly (*P* < 0.05) increasing the Bcl-2 expression as compared to DOX-administered group. Additionally, APG only administered group showed the expressions of these markers close to the control group (Fig. 3).

### Effect of DOX and APG on inflammatory indices

**Figure 4:**
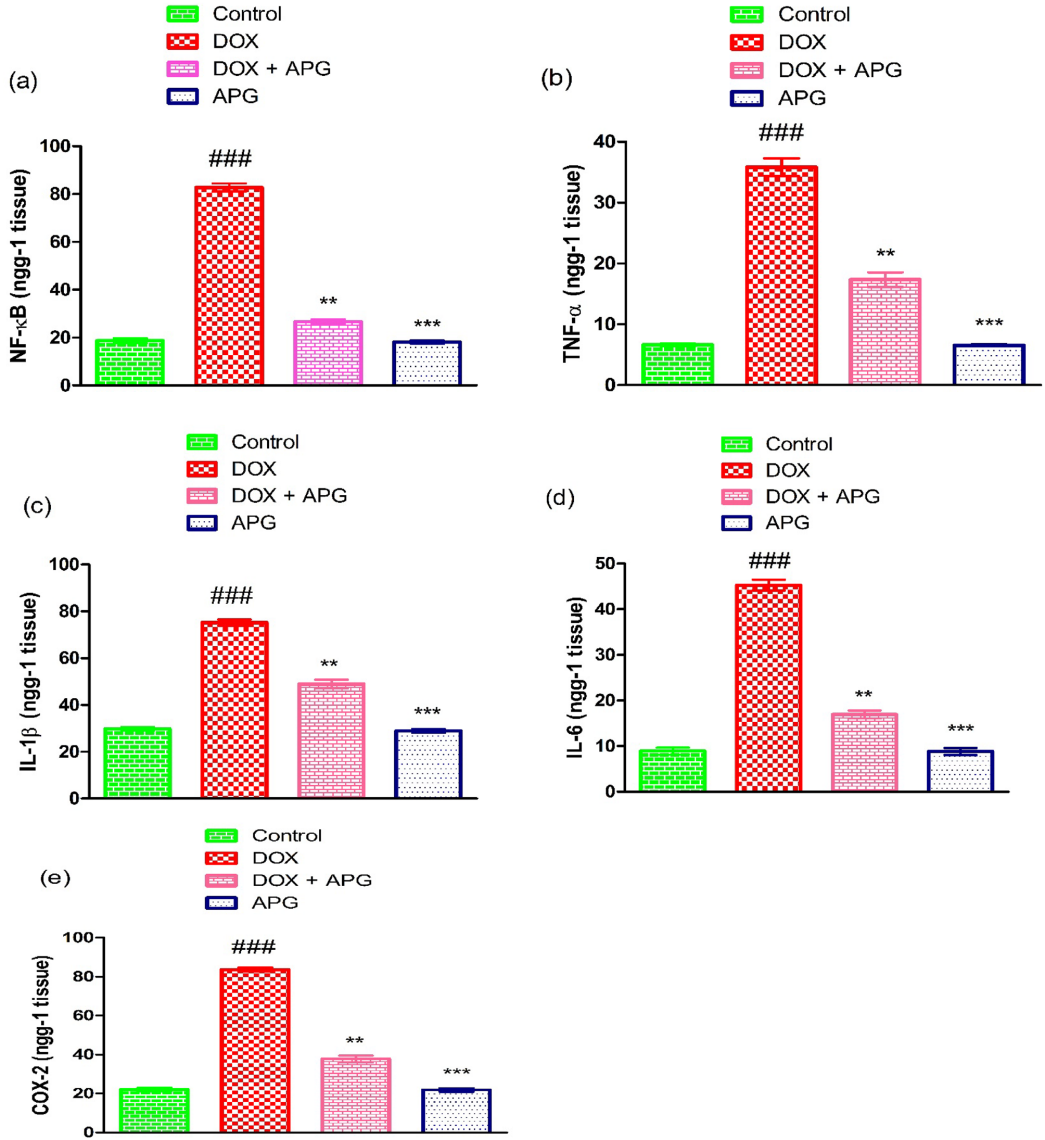
Effect of DOX and APG on the level of (**a**) NF-κB, (**b**) TNF-α, (**c**) IL-1β, (**d**) IL-6 and (**e**) COX-2. The bars are based on mean ± SEM values. Significant differences are displayed as ^###^*P* < 0.001 compared to control group; ^**^P < 0.01 compared to DOX-administered group, ^***^P < 0.001 compared to control group.

DOX administration significantly (*P* < 0.001) increased the inflammatory marker level such as, TNF-α, IL-6, NF-kB, IL-1β and COX-2 activity in comparison to control animals. However, the co-administration of APG with DOX significantly (*P* < 0.01) decreased the level of inflammatory indices as compared to DOX-administered group. Additionally, the level of inflammatory indices in APG alone administered group were similar to the control group (Fig. 4).

### Effect of DOX and APG on testicular histopathology

**Figure 5:**
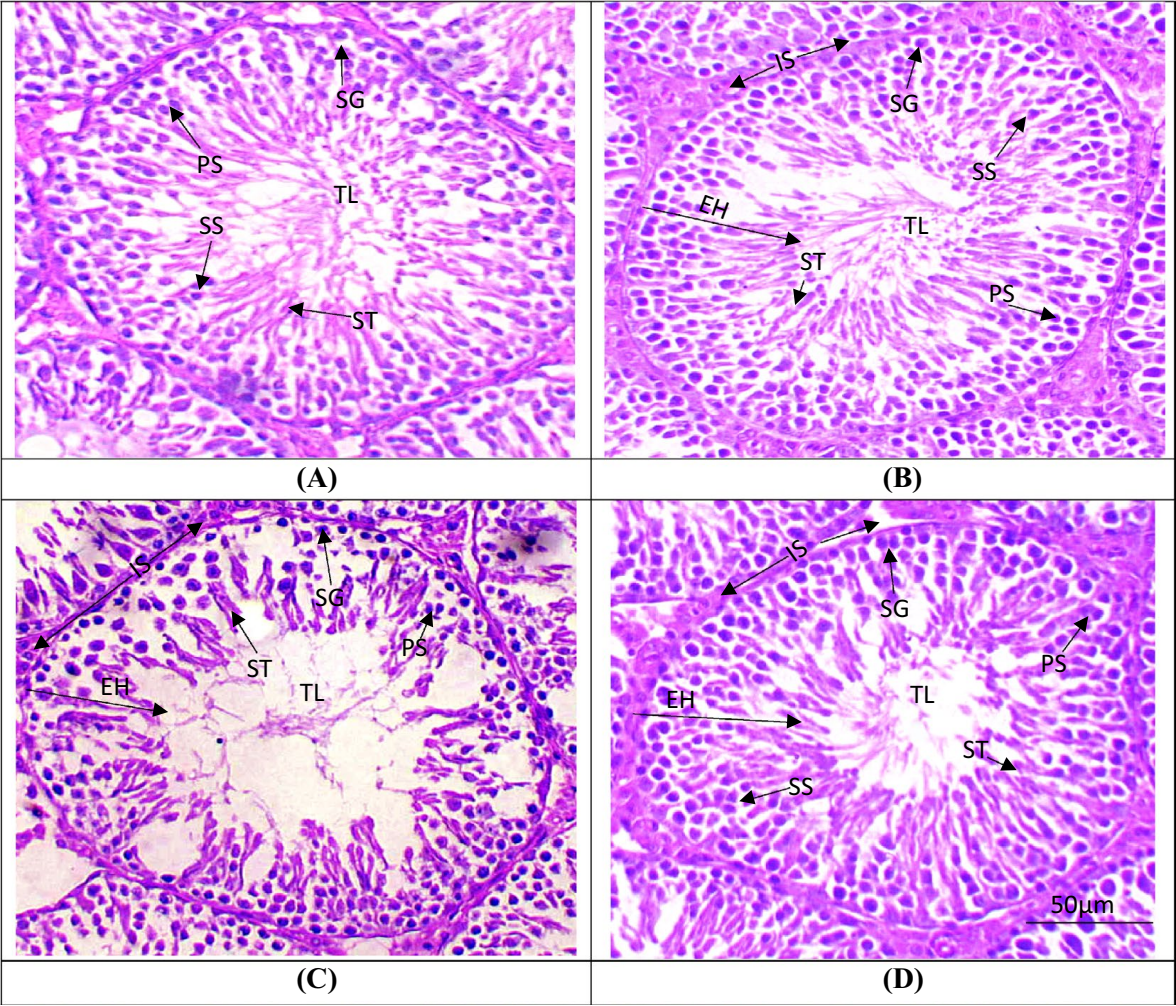
Micro-images of the adult albino rat testicles (H&E, 400X): (**A**) Control group presenting impenetrable germinal epithelium exhibiting germ cells & tapered luminal area with sperms; (**B**) APG treated group showing compacted seminiferous tubules with less IS and luminal part filled with germ cells; (**C**) DOX exposed group showing sloughing of the epithelial layer, vacant lumen & degenerated area of IS; (**D**) DOX + APG co-administered group demonstrating renovation in epithelial part as well as TL filled with ST and recovered deteriorated IS. DOX: Doxorubicin; APG: Apigetrin TL: Tubular lumen; EH: Seminiferous epithelial height; IS: Interstitial spaces; SG: Spermatogonia; PS: Primary spermatocytes; SS: Secondary Spermatocytes; ST: Spermatids.

DOX-intoxication induced a significant (*P* < 0.001) decrease in seminiferous tubular diameter as well as height and width of tunica propria. While a significant (*P* < 0.001) increase in the interstitial space and luminal diameter was observed as compared to the control group. Additionally, DOX treatment caused a significant (*P* < 0.001) reduction in the germ cells count. However, the co-administration of APG with DOX resulted in significant (*P* < 0.01) increase in seminiferous tubular diameter as well as height and width of tunica propria. Conversely, a significant (*P* < 0.01) decrease in the luminal diameter and interstitial spaces was observed as compared to DOX administered group. Furthermore, histopathological profile in only APG administered group was similar to the control group (Table 4, Fig. 5).

**Table 4:** Effect of DOX and APG on testicular histopathology.

Parameters	Groups			
	Control	DOX	DOX + APG	APG
Interstitial spaces (μm)	6.55 ± 0.68	73.90 ± 1.85^###^	19.48 ± 1.49^b^**	6.52 ± 0.67***
Tunica propria (μm)	85.63 ± 1.19	15.40 ± 1.23^###^	64.34 ± 1.52**	86.53 ± 1.62***
Seminiferous tubules diameter (μm)	372.44 ± 5.17	138.41 ± 5.12^###^	241.06 ± 6.08**	375.21 ± 6.09***
Seminiferous tubule epithelial height (μm)	74.44 ± 1.97	28.46 ± 1.81^###^	73.57 ± 1.84**	73.39 ± 2.59***
Tubular lumen (μm)	32.68 ± 0.97	97.51 ± 3.06^###^	57.14 ± 1.57**	32.17 ± 0.33***
Spermatogonia (n)	65.59 ± 1.32	24.80 ± 0.76^###^	54.35 ± 1.05**	66.33 ± 1.15***
Primary spermatocytes (n)	55.99 ± 1.83	19.56 ± 1.62^###^	44.22 ± 1.87**	58.61 ± 2.74***
Secondary spermatocytes (n)	46.77 ± 2.55	15.74 ± 1.58^###^	34.78 ± 1.74**	48.64 ± 3.18***
Spermatids (n)	63.73 ± 0.90	24.95 ± 1.84^###^	37.3 ± 15.85**	64.55 ± 1.22***

Values based on mean ± SEM values. Significant differences are displayed as ^###^*P* < 0.001 compared to control group; **P < 0.01 compared to DOX-administered group, ***P < 0.001 compared to control group.

## Discussion

DOX is a powerful, commonly prescribed and a strong anti-cancer drug. However, its clinical application is limited due to its potential side effects such as, cardiotoxicity^37^, hepatotoxicity^38^, nephrotoxicity^39^ and testicular toxicities^40^. DOX intoxication is reported to disturb hormone levels, sperm parameters, apoptotic markers, antioxidant enzymes, steroidogenic enzymes and testicular histopathology^41^. DOX exposure may damage the quantity and quality of sperm in adults^42^. It is reported that natural anti-oxidant can be used to treat OS-related disorders^43,44^. Apigetrin (APG) is a natural flavonoid that is present in the leaves, seeds and fruits of *Teucrium gnaphalodes, Stachys tibetica Vatke,* and *Scutellaria baicalensis Georgi*^45^. APG shows anti-hyperglycemic, anti-oxidant, anti-carcinogenic, anti-inflammatory and anti-viral effects^46^. Therefore, the present research was planned to assess the therapeutic role of APG in mitigating DOX-induced testicular damage in rats.

DOX exposure significantly reduced the activities of antioxidant enzymes (CAT, GSR, SOD and GPx), while increasing the levels of ROS and MDA. ROS includes superoxide radicals (O_2_^–^), hydroxyl radicals (OH^–^) and hydrogen peroxide (H_2_O_2_), which are metabolic byproducts that may cause harm to cells of the body. Our results are further supported by the previous study in which it was reported that DOX showed the potential to induce OS in rats and reduced the activities of anti-oxidant enzymes^47^. The production of ROS leads to OS that ultimately damage the quality and quantity of sperms^48^. Elevated ROS production impairs spermatozoa's anti-oxidant defense mechanism^49^. CAT is one of the major antioxidant enzyme that plays a substantial part in H_2_O_2_ catabolism^50^. It separates NADPH oxidase-generated superoxide radicals and safeguards the spermatozoa from oxidative damage^51^. SOD transforms superoxide into H_2_O_2_ and O_2_^52^. GSR facilitates the transformation of GSH from its reduced state to its oxidized state^53^. GPx helps in lowering the lipid peroxide (LP) and H_2_O_2_ concentrations and plays an important role in reducing OS^54^. CAT, GPx, SOD and GST are anti-oxidant enzymes that are pivotal in alleviating ROS, OS and eventually reduce the damage^55^. MDA is a byproduct of ROS-induced LP and it is used as a biomarker of LP and OS^56^. In order to minimize OS, plant sources can also be used as a supplement^57^. The co-administration of APG with DOX lowered the OS in rat testes and increased the anti-oxidant enzymes activities, besides lessened the level of ROS and MDA due to its anti-oxidant potential. Moreover, our results are further endorsed by the study of Kale et al.^58^ who stated that double bonds between C2 and C3 in the structure of APG are responsible for its anti-oxidant activity.

DOX intoxication significantly reduced the sperm viability, number and motility. Besides dead sperm count and sperm tail, head and mid-piece abnormalities were increased in the DOX exposed rats. Our findings are in line with the research of Ahmad et al.^59^ who stated that DOX administration resulted in reduced sperm count, impaired motility, induced spermatotoxicity, and spermatogenic failure. Sperm quality is a crucial factor in ascertaining male reproductive capability, where the primary indicators are sperm count and mortality rate. Reduced sperm count can be due to the delayed maturation and abnormal germ cell proliferation^60^. According to previous literature, large amount of polyunsaturated fatty acids (PUFAs) make spermatozoa susceptible to OS. The motility of sperm and the integrity of sperm plasma membrane depend on the PUFAs^61^. The fluidity of sperm plasma membrane and the process of spermatogenesis are severely affected by OS^62^. Furthermore, OS can directly affect sperm cell mitochondria and results in reduced ATP production, flagellar activity and eventually leads to apoptotic death and immobility^63^. However, the supplementation of APG mitigated all these DOX prompted impairments due to its ROS scavenging nature.

DOX exposure significantly reduced the expressions of 3β-HSD, 17β-HSD and StAR. These enzymes play a pivotal role in the production of steroid hormones^64^. StAR acts as a regulatory protein during the production of testosterone and it plays a key role in the transportation of cholesterol inside the mitochondrial membrane^65,66^. 17β-HSD and 3β-HSD catalyzes the conversion of cholesterol to testosterone^67^, while lowered expressions of these enzymes, decrease the level of testosterone^68^. Nevertheless, the supplementation of APG significantly increased the steroidogenic enzymes expression and restored the level of testosterone. Moreover, it is stated that flavonoids have chemical structure similar to cholesterol that could regulate the production of androgens^69^.

In the current investigation, DOX intoxication significantly decreased the level of plasma testosterone, FSH and LH. According to the study conducted by Mustafa et al.^22^ DOX possesses the potential to reduce the hormonal level in rats. Spermatogenesis is controlled by a proper ratio of testosterone, FSH and LH^70^. The production of testosterone is crucial for the reproductive health of male^71^. Both FSH and LH are vital to control spermatogenesis^72^. LH induces LCs to generate testosterone, besides FSH encourages Sertoli cell proliferation^73^. However, the supplementation of APG improved the levels of these hormones and eventually recovered the spermatogenesis. Our findings are supported by the research of Agrawal^74^ who stated that flavonoids have the potential to control the production of hormones i.e., estrogen and androgen.

DOX exposure augmented the expressions of Bax and Caspase-3, while reducing Bcl-2 expression. Bcl-2 acts as anti-apoptotic protein that safeguards the cells from apoptotic death, Bax accelerates the process of apoptosis^75^. A reduction in Bcl-2 and elevation in Bax expression induce significant alteration in mitochondrial membrane permeability, which eventually stimulates the liberation of cytochrome C into the cytoplasm^76^. Caspase-3 belongs to the cysteine aspartic protease family and plays a pivotal role in initiating and executing cellular apoptosis^77^. Caspase-3 is activated by the augmented level of cytochrome C that leads to cell death or apoptosis^78^. However, the administration of APG augmented the Bcl-2 expression, whereas lowered the expression of Caspase-3 and Bax due to its anti-apoptotic nature.

DOX administration significantly increased the levels of inflammatory markers, such as TNF-α, IL-1β, NF-kB, IL-6 and COX-2 activity. The results of our research are supported by the research of Hu et al.^79^ who stated that DOX exposure augmented the level of inflammatory markers in the testicular tissue of rats. Inflammation is one of the major cause of male infertility^80^. NF-κB instigation is pivotal for the expression of proinflammatory cytokines (IL-6, TNF-α, COX-2 and IL-1β) that are related with severe inflammation and other ROS associated disorders^81^. COX-2 is also an inflammatory marker that remarkably contributes to inflammation^82^. However, the administration of APG with DOX resulted in a considerable reduction in inflammatory indices due to its anti-inflammatory nature. Moreover, our results are further supported by the previous study in which it was reported that APG has the potential to attenuate lipopolysaccharide induced inflammation in rats^23^.

DOX exposure significantly increased the diameter of tubular lumen and interstitial spaces. Besides, it reduced the seminiferous tubular diameter and epithelial height, tunica propria width and germ cells count. The findings of our current histopathological investigation are in line with an earlier study, in which DOX-intoxication reduced seminiferous tubular diameter as well as germ cells number. It was also reported that DOX-intoxication reduced the testosterone level, which ultimately reduced the germ cells count and induced histopathological damages^83^. According to Adejuwon et al.^84^ an imbalance between pro-oxidants and anti-oxidants results in the overproduction of ROS that leads to histopathological damage. The disruption of steroidogenic enzymes expression as well as a decrease in testosterone level is one of the major reasons of spermatogenesis failure^85^. However, APG treatment considerably augmented the germ cells count and restored all the testicular anomalies due to its anti-apoptotic, anti-oxidative, anti-inflammatory and androgenic properties.

## Conclusion

The findings of the current study indicated that APG exhibited excellent ameliorative role against DOX-induced testicular damage by reducing the oxidative stress. APG administration significantly regulated the activities of anti-oxidant enzymes, the levels of MDA ROS and inflammatory indices, the expressions of steroidogenic enzymes, apoptotic and anti-apoptotic proteins as well as histological profile of testes. Therefore, our findings revealed that APG may be used to cure testicular damage. However, this study was perform by using rats as animal model so, its clinical trials on humans are recommended in future.

## Data Availability

All the data is contained in the manuscript**.**
